# Lethal and severe violence: Characterizing Swedish female offenders with and without a severe mental disorder

**DOI:** 10.3389/fpsyt.2023.1143936

**Published:** 2023-04-06

**Authors:** Karin Trägårdh, Malin Hildebrand Karlén, Peter Andiné, Thomas Nilsson

**Affiliations:** ^1^Forensic Psychiatric Clinic, Sahlgrenska University Hospital, Gothenburg, Sweden; ^2^Centre for Ethics, Law and Mental Health, Institute of Neuroscience and Physiology, Sahlgrenska Academy, University of Gothenburg, Gothenburg, Sweden; ^3^Department of Forensic Psychiatry, National Board of Forensic Medicine, Gothenburg, Sweden; ^4^Department of Psychology, University of Gothenburg, Gothenburg, Sweden

**Keywords:** lethal violence, severe violence, homicide, female offender, mentally disordered violent offender

## Abstract

**Aim:**

In studies on lethal and severe violence, male offenders have historically been in focus while female offenders, in comparison, have often been excluded. In this study, we aimed to characterize female violent offenders and compared those with and without a severe mental disorder (SMD).

**Method:**

All females charged with lethal or attempted lethal violence, who had undergone forensic psychiatric investigations (FPI) in Sweden between 2000 and 2014, constituting the two groups SMD (*n* = 84) and no SMD (*n* = 91), were included. Information from their FPI reports and court verdicts was collected regarding background and demographics, mental health, substance use, and crime characteristics.

**Results:**

Overall, both groups were often unemployed, previously victimized within close relations, had psychiatric health issues, and more than half of them had previously attempted suicide. Specifically, the SMD group more often had psychotic disorders, had attempted homicide-suicide (at the time of the crime), and had children or friends/acquaintances as victims. The no-SMD group more often manifested patterns of anxiety, personality disorders, and substance use disorders compared to the SMD group. The no-SMD group also differed from the SMD group by more often having a previous criminal record, being charged with lethal index violence, having male adult intimate partners/ex-intimate partners as victims who had abused the offender, and both offender and victim had more often been under the influence of a substance.

**Conclusion:**

Female offenders of lethal and severe violence had a high prevalence of previous violent victimization which should be considered in forensic assessment and treatment regardless of the offender’s SMD status. However, more focus on substance use disorders and intimate partner relations appears relevant for females without an SMD. Contrary to that, early interventions regarding psychotic processes are probably a helpful preventive measure for females with an SMD. In sum, the heterogeneity of female offenders of lethal and severe violence emphasizes the necessity of developing nuanced interventions to meet their rehabilitative needs as well as the requirements of community protection.

## 1. Introduction

About 470,000 homicide victims were reported globally in both 2015 (1) and 2017 (2), most commonly with male victims as well as male offenders, with severe consequences on both an individual and societal level (e.g., death and serious injuries, loss of relatives, as well as psychological, social, and economic stress). Female offenders are responsible for approximately 10% of homicides overall (3, 4), but approximately 30–50% of homicides with child victims (5–8). Despite this, research on female lethal and severe violent offenders (hereafter: female offenders) has been relatively scarce. In Sweden, female offenders (as well as their male counterparts) usually follow two separate legal trajectories, due to the division between those who after a forensic psychiatric investigation (FPI) are considered suffering from what in Swedish law is termed a severe mental disorder (SMD) (9), and those who are not. The former group is almost always sentenced to forensic psychiatric care, while the latter (i.e., no-SMD) is almost always sentenced to prison. Given the lack of research on female offenders and the grave implications of the SMD decisions for them, it is essential to understand both what characterizes the group as a whole and whether specific offender characteristics separate those with an SMD from those without. This would, if so, have consequences for both future risk management and rehabilitation as well as the development of preventive measures.

Further, aggression and violence, central concepts within this study, are complex and challenging to define and understand. Most previous studies on this subject, as demonstrated below, have based previous and current violence on registered sentences for violent crimes. Nevertheless, these concepts have also been defined from different theoretical angles, such as the general aggression model (10–12), theories on reactive versus instrumental aggression/violence (13), and behavioral and neurobiological perspectives on aggression in women (14). Hence, based on these perspectives, aggression is defined as actions with the intent to harm other targets. In addition, violence is defined as aggressive actions directed toward others with the intent to physically harm (incl. attempt, threat, and actual damage) in a more severe manner (e.g., leading to physical injury or death). Furthermore, how to understand and explain how aggression and violence occur and are acted out varies between different perspectives. However, there appears to be a consensus that these behaviors have a multifactorial and complex basis. For instance, the general aggression model emphasizes episodic processes regarding person and situation (based on the individual’s personality, in turn, influenced by biological and environmental modifiers), current internal states (i.e., cognitive, affective, and arousal routes), and lastly outcomes of the fundamental appraisal and decision processes, which further affects the individuals thoughtful or impulsive action (10, 11).

Previous studies on female offenders have often focused on specific subgroups, such as female offenders having specific victim groups, for example, intimate partner homicide (15–18), or child victims (19, 20), or specific offender characteristics such as substance abuse (21, 22) or mental disorders (23–25). Also, the sample sizes in previous studies have often been small (*n* < 20) (e.g., 15, 18, 20, 26), which limits the possibility to generalize findings. This focus on specific subgroups in a field characterized by multifaceted heterogeneity and different jurisdictions, often with small sample sizes, has resulted in highly specific knowledge limited to demarcated subgroups of female offenders. Thus, specific subgroup patterns have been identified, yet generally based on restricted samples, but more general patterns have not. Due to this situation, there is still a lack of knowledge regarding general factors and problem areas characterizing female offenders. For the present study, research that has focused on (a) demographic and background factors, (b) mental health, (c) substance use/abuse, and (d) crime characteristics among females who have committed lethal and severe violent crimes is relevant to consider.

Basic demographic factors associated with female offenders are: low educational level (15, 19, 23, 27, 28); unemployment (18, 27, 29); adverse childhood experiences [i.e., mental, physical, and sexual abuse and/or domestic dysfunction (30)] (20, 27, 31); life-time maltreatment/trauma victimization (e.g., mental, physical, and sexual abuse) (26), as well as having tried to get help during the 6 months preceding the crime (29). The high level of victimization among female offenders has implications for prevention, treatment, and rehabilitation. When working with this group, a specific focus on trauma treatment has been suggested as a preventive measure (32–34). Regarding civil status, various results have been obtained, with some studies showing that female offenders often are married/within intimate partner relations (16, 27, 29), while other studies have found that they are seldom in a relationship (23, 26, 34). This discrepancy could, at least in part, be an effect of differences in sample selection, but it creates a contradictory picture that requires access to population-based samples.

Previous research on mental health among female offenders often separates those with severe mental illness (generally treated within forensic mental health services) from those without such an illness (treated within the correctional system). Among female offenders within forensic psychiatric care, previous psychiatric disorders are common, where the clinical picture commonly is dominated by psychotic disorders (23) and mood disorders (20). Prior research on incarcerated female offenders on the other hand also showed a high prevalence of mood disorders, and quite often borderline and antisocial personality disorders (31), as well as various developmental disorders (e.g., intellectual and/or learning disabilities, and/or ADHD) (34). The somewhat overlapping picture of mental illness independent of the type of sanction might be a result of differences in jurisdiction between different countries in combination with small sample sizes. Furthermore, previous mental healthcare contact and suicide attempts were also common among female offenders (27). Regarding cognitive functioning, female offenders, at least within a prison population, in general, performed within the lower part of the normal range, in combination with a particularly low verbal ability (34). Some studies have also found a high prevalence of traumatic brain injuries (e.g., frontal lobe damage) among female offenders (33).

In general, previous research indicates that most female offenders have a history of substance abuse (27, 35). This finding is also independent of whether the focus is on forensic psychiatric care samples (21, 26), or prison samples (34). Though, even if the positive correlation between substance abuse and crime appears predominant, there are contradictory findings regarding intoxication at the time of the crime. For instance, prior studies regarding female homicide offenders within prison (36), as well as intimate partner homicide offenders (18), have shown that a majority of them were intoxicated at the time of their crime. However, other findings point to variations within different samples, for example, only a minority of the female violent offenders within forensic psychiatry were intoxicated during their crime (21), and the female violent (but non-homicide) compared to homicide offenders seem to have more severe substance use problems, including intoxication at the time of the crime (26). Further, even though the offenders within the forensic psychiatric sample with substance abuse had shown an earlier debut in criminality with more frequent previous convictions, substance abuse *per se* did not predict reoffending for this subgroup (21).

Generally, studies have shown relatively limited criminal records for female offenders (especially compared to male offenders) (26, 27, 37), which was even more pronounced for female offenders of lethal violence within the primary family compared to such violence within a non-family context (38). Throughout the world, victim-characteristics of female homicide offenders consistently are found to be relatives (23, 38, 39), most commonly male (40) intimate partners/ex-intimate partners (41), followed by other family members, including children (29), and thereafter acquaintances/friends (34, 42). Furthermore, in the case of intimate partner homicide, the female offender had often been victimized (either previously and/or at the time of the homicide) by the male victim (18). Moreover, the lethal crime typically took place within a homelike environment (20, 39), and regarding the type of violence, sharp/knife violence was the most common method for female homicide offenders (20, 42), whereas asphyxia has been found to be most common with child victims (5, 6).

Based on this heterogenic and piecemeal picture of female offenders, there is a need to establish a broader and more holistic characterization of this group, focusing on specific areas where results from previous research have been contradictory or questionable such as mental health, substance use, and victim-offender relations. Given this background, our general aim is to describe and compare female offenders of lethal and attempted lethal violence, who had an SMD versus those who did not have an SMD. More specifically, the aims of this study were (i) to characterize female offenders who had undergone an FPI due to lethal and severe violence, and (ii) compare female offenders with versus without an SMD regarding; (1) background and demographics, (2) mental health, (3) substance use/abuse, as well as (4) crime characteristics.

## 2. Methods

### 2.1. Legal setting and the concept of FPIs

According to Swedish jurisdiction (43), all offenders are held responsible for their actions even if their mental state is questioned. They can, if their mental problems are severe enough, be judged to suffer from an SMD, a medico-legal concept comprising serious mental health problems (e.g., major mental illnesses such as psychotic syndromes, severe depression with suicidal ideation, severe developmental syndromes, and severe personality syndromes with either extensive compulsivity or psychotic reactions). To clarify, an SMD often comprises a psychotic disorder, but not always. An SMD can also consist of a personality disorder in combination with low functioning if it is considered severe enough, even if this is relatively rare. Furthermore, an offender can have a psychotic disorder and at the same time being assessed not having an SMD at the time of the crime, even if this also rarely occurs. The latter could for example be the case when an individual with a psychotic disorder shows no psychotic symptoms at the time of the crime due to medication. Another example is when an offender shows psychotic symptoms, but this is assessed as being caused by the individual itself (e.g., self-induced intoxication or a lack of compliance concerning medication). When found guilty and suffering from an SMD, the penal code (43) prescribes, as a rule, compulsory forensic psychiatric care instead of a prison sentence. To determine whether a suspect suffers from an SMD, an FPI is performed by the Swedish National Board of Forensic Medicine (a state authority) when ordered by the court. An FPI generally takes place for a maximum of 4 weeks and is performed by a team of forensic professionals working together. Generally, the forensic professionals (i.e., a psychiatrist, psychologist, and social worker) have access to all documents regarding the offender’s historical and present contacts with different community institutions (e.g., social services, healthcare, education, and previous correctional sanctions), as well as the preliminary police investigation on the present crime. In addition, the forensic professionals use different methods such as interviews, tests, and structured instruments within their investigation to increase the accuracy of the SMD assessment. Hence, the amount of information on which the FPIs are based is extensive and generally rich in detail, making them (at least in principle) the most comprehensive kind of psychiatric assessment possible in Sweden (for more information on the Swedish legislation, see 9, 44, regarding FPI, see 45).

### 2.2. Procedure, study population, and data sampling

In Sweden, a majority of female offenders charged with lethal violence (incl. attempted) are subjected to an FPI (ranging from 7 to 8 out of 10; 46–49). This circumstance was used for data collection, why this descriptive cross-sectional study reviewed all Swedish FPIs from the years 2000–2014, together with the associated court verdicts, regarding women who were suspects of/charged with lethal or attempted lethal violence. The FPIs were systematically reviewed, and the collected information was transferred to a research protocol.^1^ The resolution in the protocol was very high, so in order to get a better overview, certain variables were merged before they were analyzed. This process of coding data from FPIs and associated information was done by licensed clinical forensic psychologists (mainly KT, MHK, and TN).

#### 2.2.1. Sample selection

All women who had undergone an FPI between 2000 and 2014 and were charged with one of the following crimes: murder, voluntary manslaughter, attempted murder, attempted voluntary manslaughter, assault combined with involuntary manslaughter, infanticide, and conspiracy/instigation/preparation to murder or attempted murder, were identified. A total of 178 individuals first met the inclusion criteria during the period in question. However, three cases (1.7%) were excluded due to receiving an acquittal in court (i.e., based on being considered not guilty), rendering a total of 175 cases. Given that a majority of woman that has perpetrated lethal and severe violence undergoes an FPI, these 175 cases represent a majority of all female offenders that had perpetrated the offenses in question during this period. However, for obvious reasons, female offenders who had committed homicide-suicide in connection to their crime (about 2.5% of female homicide offenders with adult victims, and 30% with child victims, during this period (see 6)) were not included in this study. The study was reviewed and approved by the Regional Ethical Board in Gothenburg (registration number: 788–14).

### 2.3. Instrument and variables

For all individuals included in the study, their FPIs and associated court verdicts were systematically reviewed. Information was compiled by using a research protocol^2^ covering the following areas: psychosocial background and demographics, mental health, substance use/abuse (incl. age of onset and severe negative consequences),^3^ presence of risk factors for severe criminality, previous sentences, sanctions (i.e., custody or forensic psychiatric care), offense behavior, and victim-offender relationship. Areas that are presented in the present study consisted of the following specific sub-areas: Background and demographics (e.g., date of birth, social status, adverse experiences in childhood and adulthood [e.g. offender abused within family, abused defined as physical, sexual, and/or verbal violence/aggression], education and employment); Mental health (e.g., previous child and adolescent institutional care, psychiatric care [both in- and out-patient], and psychiatric diagnoses [both previous and at the time of the FPI]); Substance use/abuse (i.e., history of substance use/abuse, previous substance use/abuse treatment services and substance use disorder diagnoses [both previous and at the time of the FPI]); Crime characteristics (e.g., history of registered criminality according to previous sentences [covering violent crime, property crime, drug crime, financial crime, aggravated traffic crime, and restraining orders], current crime [e.g., lethal vs. attempted lethal violence, and offender behavior characteristics such as method of violence, offender under the influence of substance, offender abused victim, abused defined as physical, sexual, and/or verbal violence/aggression], victim-offender relationship and victim behavior characteristics such as victim under the influence of substance, victim abused the offender, abused defined as physical, sexual, and/or verbal violence/aggression). Information about diagnoses of mental health was based on the presence of diagnoses assessed and documented in the FPI, according to the Diagnostic and Statistical Manual of Mental Disorders DSM-IV. In several cases participants fulfilled criteria for more than one diagnosis.

### 2.4. Statistical analyses

Analyses were performed on background and demographics, mental health, substance use/abuse, and crime characteristics. General descriptions for the entire group were obtained and comparisons between the group considered to have an SMD versus the group who had not, henceforth referred to as the “SMD group” versus the “no-SMD group.” To compare differences between these two groups, a *χ*^2^-test or Fisher’s exact test (the latter when the expected values in any of the cells of the table were below five) was used for categorical variables, and Student’s t-test for continuous variables. Due to missing data, there was some variation in the number of subjects in the separate analyses, whereas all reported percentages were valid (i.e., missing cases not included). Confidence intervals (95% CI) and two-tailed probability values, α-level = 0.05, as well as α-level corrected by the Bonferroni method (i.e., 0.0009 rounded to 0.001), were reported. Effect sizes used were Odds ratio and Cramer’s V for categorical variables and Cohen’s D for continuous variables. Effect sizes were reported according to the following recommendations: For Odds ratio, same odds to display the variable for the SMD and the no-SMD group = 1.0 (or close to 1.0), greater odds of displaying the variable among the SMD group vs. the no-SMD group >1.0, lower odds of displaying the variable among the SMD group vs. the no-SMD group <1.0; For Cramer’s V (depending on the *df*), small effect ranging = 0.04–0.10, medium effect ranging = 0.13–0.30, and large effect ranging = 0.22–0.50; For Cohen’s D, small effect = 0.20, medium effect = 0.50, and large effect = 0.80 (50).

For interrater reliability analyses, intraclass correlation coefficient (ICC) was used (51). Two-way mixed effects model, single rater type, and absolute agreement as definition, were used. Variables that required at least a certain amount of subjective interpretation were chosen for this reliability analysis. A total of 50 variables were included in this analysis and stratified into groups depending on their ICC values (poor reliability <0.50; moderate reliability = 0.50–0.75; good reliability = 0.75–0.90; and excellent reliability >0.90). Ratings of variables were divided between four raters, resulting in 20% of the total sample rated by two persons. All in all, a majority of the analyzed variables presented in this study showed ICC values indicating excellent interrater reliability (54.0%), good interrater reliability (24.0%), and moderate interrater reliability (6.0%) values (for more detailed information, see Supplementary Table S1 – ICC). All analyses were performed in SPSS version 26 (SPSS Inc., Chicago, IL, United States).

## 3. Results

### 3.1. Background and demographics

The 175 female offenders included in this study and originating from the 15-year inclusion period consisted of 84 offenders (48%) with an SMD (the SMD group) and 91 offenders (52%) without an SMD (the no-SMD group; Table 1). Overall, the majority were neither established on the job market (53.1%) nor in employment/studies at the time of the crime (71.5%). Regarding civil status at the time of the crime, a large part lived with an intimate partner (40.7%), but the majority were single (46.0%), whereof a large majority had previously lived with an intimate partner. Additionally, a majority of the offenders had been abused within the primary family during their childhood/adolescence (<18 years; 56.5%), and many had also experienced substance abuse among their guardians (48.1%). This pattern was also seen in adulthood (>18 years) where 68.5% had been abused by an intimate partner, not seldom in combination with substance abuse by cohabitants (e.g., partners, friends; 52.1%). Despite these observed variations, there were no significant differences between the SMD- versus no-SMD group regarding background factors and demographics.

**Table 1:** Background and demographics regarding women, with and without a severe mental disorder^#^ (SMD), who were charged with lethal or attempted lethal violence and examined according to a court-ordered forensic psychiatric investigation in Sweden, carried out between 2000 and 2014.

	All offenders (*n* = 175)	SMD group (*n* = 84)	No-SMD group (*n* = 91)	*p* value (*χ*^2^)	Effect size
Offender, age in years				0.132	0.229^†††^
Mean (SD)	35.7 (11.9)	34.2 (11.5)	37.0 (12.3)		
Country of birth, born in Sweden, *n* (%)				0.591	0.842 (0.450–1.577)^†^
No	59 (33.7%)	30 (35.7%)	29 (31.9%)		
Yes	116 (66.3%)	54 (64.3%)	62 (68.1%)		
Civil status, *n* (%)				0.077	0.221^††^
Living with family of origin	18 (10.5%)	10 (12.0%)	8 (9.0%)		
Living with friends	5 (2.9%)	3 (3.6%)	2 (2.2%)		
Single, lived alone during adulthood	18 (10.5%)	13 (15.7%)	5 (5.6%)		
Single, previously lived with intimate partner	61 (35.5%)	31 (37.3%)	30 (33.7%)		
Living with IP	70 (40.7%)	26 (31.3%)	44 (49.4%)		
Custody of child, *n* (%)				0.658	1.165 (0.591–2.298)^†^
No	129 (74.1%)	61 (72.6%)	68 (75.6%)		
Yes	45 (25.9%)	23 (27.4%)	22 (24.4%)		
Educational level, *n* (%)				0.167	0.194^††^
Not passed elementary school	24 (14.0%)	10 (12.2%)	14 (15.7%)		
Elementary school for pupils with learning disabilities	3 (1.8%)	1 (1.2%)	2 (2.2%)		
Elementary school	57 (33.3%)	31 (37.8%)	26 (29.2%)		
Upper secondary school	66 (38.6%)	26 (31.7%)	40 (44.9%)		
College/university	21 (12.3%)	14 (17.1%)	7 (7.9%)		
Established on the job market (more than short-term and temporary employment) at some point in life, *n* (%)				0.843	0.939 (0.504–1.749)^†^
No	85 (53.1%)	41 (53.9%)	44 (52.4%)		
Yes	75 (46.9%)	35 (46.1%)	40 (47.6%)		
Employment/studies at the time of the crime, *n* (%)				0.706	0.147^††^
Student	22 (12.6%)	12 (14.3%)	10 (11.0%)		
Job, part-time	4 (2.3%)	2 (2.3%)	2 (2.2%)		
Job, full time	11 (6.3%)	4 (4.8%)	7 (7.7%)		
Unemployed	61 (34.9%)	25 (29.8%)	36 (39.6%)		
On sick leave	28 (16.0%)	14 (16.7%)	14 (15.4%)		
Activity and sickness compensation	36 (20.6%)	21 (25.0%)	15 (16.5%)		
Other	13 (7.4%)	6 (7.1%)	7 (7.7%)		
**Adverse childhood experiences (<18 years of age)**					
Substance abuse by guardians, *n* (%)				0.508	0.810 (0.433–1.514)^†^
No	82 (51.9%)	41 (54.7%)	41 (49.4%)		
Yes	76 (48.1%)	34 (45.3%)	42 (50.6%)		
Offender, abused^1^ within primary family *n* (%)				0.098	0.575 (0.298–1.111)^†^
No	64 (43.5%)	35 (50.7%)	29 (37.2%)		
Yes	83 (56.5%)	34 (49.3%)	49 (62.8%)		
Offender, abused^1^ outside primary family *n* (%)				0.192	0.628 (0.311–1.266)^†^
No	90 (64.7%)	49 (70.0%)	41 (59.4%)		
Yes	49 (35.3%)	21 (30.0%)	28 (40.6%)		
**Adverse adulthood experiences (>18 years of age)**					
Substance abuse by cohabitants, *n* (%)				0.326	0.716 (0.368–1.396)^†^
No	67 (47.9%)	34 (52.3%)	33 (44.0%)		
Yes	73 (52.1%)	31 (47.7%)	42 (56.0%)		
Offender, abused^1^ by intimate partner, *n* (%)				0.075	0.509 (0.241–1.077)^†^
No	41 (31.5%)	22 (40.0%)	19 (25.3%)		
Yes	89 (68.5%)	33 (60.0%)	56 (74.7%)		
Offender, abused^1^ by other than intimate partner, *n* (%)				0.689	0.847 (0.377–1.907)^†^
No	57 (58.8%)	28 (60.9%)	29 (56.9%)		
Yes	40 (41.2%)	18 (39.1%)	22 (43.1%)		
					

^#^The concept of a severe mental disorder is the Swedish legal term for when an offender according to a forensic psychiatric investigation is judged to fulfill criteria for compulsory forensic psychiatric care instead of a prison sanction. The method chosen to calculate the effect size, indicated by a specific symbol as shown below, depends on the statistical test we have performed. ^†^Odds ratio, 95% confidence interval. ^††^Cramer’s V. ^†††^Cohen’s D. ^1^Abused, defined as physical, sexual, and/or verbal violence/aggression.

### 3.2. Mental health characteristics

In total, a majority of the 175 female offenders had previously received psychiatric diagnoses (DSM-IV, Axel I [excl. Individuals diagnosed with only substance use disorders] and Axel II; 71.1%), psychiatric care (81.7%), and/or attempted suicide (60.6%; Table 2). There were no significant differences between the SMD and the no-SMD group regarding these variables. Although a clear majority of all offenders had some type of psychiatric diagnosis(es) (Axel I [excl. Individuals diagnosed with only substance use disorders] Axel II, and Axel III) at the time of the FPI (90.9%), there was a significant difference where the odds of having a diagnosis among the SMD group were greater compared to the no-SMD group (100.0% vs. 82.4%; OR 2.120 [1.798–2.499]). While there were no significant differences between the two groups regarding most of the diagnoses, the odds of having schizophrenia or other psychotic disorders among the SMD group were unsurprisingly greater compared to the no-SMD group (47.6% vs. 1.1%; OR 81.818 [10.888–614.802]). Contrary to that, personality disorders were less frequent in the SMD group compared to the no-SMD group (25.0% vs. 45.1%; OR 0.407 [0.214–0.774]), as was anxiety disorders (13.1% vs. 27.5%; OR 0.398 [0.182–0.871]), although not significant according to the Bonferroni-corrected significance level.

**Table 2:** Mental health characteristics, grouped in agreement with DSM-IV overarching categories, regarding women, with and without a severe mental disorder^#^ (SMD), who were charged with lethal or attempted lethal violence and examined according to a court ordered forensic psychiatric investigation in Sweden, carried out between 2000 and 2014.

	All offenders (*n* = 175)	SMD group (*n* = 84)	No-SMD group (*n* = 91)	*P* value (*χ*^2^)	Effect size
**Previous care and mental health**					
Child- and adolescent institutional care, *n* (%)				0.470	1.318 (0.622–2.795)^†^
No	139 (80.3%)	64 (78.0%)	75 (82.4%)		
Yes	34 (19.7%)	18 (22.0%)	16 (17.6%)		
Psychiatric care, *n* (%)				0.188	1.690 (0.769–3.714)^†^
No	32 (18.3%)	12 (14.3%)	20 (22.0%)		
Yes	143 (81.7%)	72 (85.7%)	71 (78.0%)		
Suicide attempt, *n* (%)				0.879	0.952 (0.504–1.797)^†^
No	63 (39.4%)	30 (40.0%)	33 (38.8%)		
Yes	97 (60.6%)	45 (60.0%)	52 (61.2%)		
Psychiatric diagnoses^1^ [DSM-IV Axel I^2^ and/or II, *n* (%)]				0.077	1.828 (0.934–3.580)^†^
No	50 (28.9%)	19 (22.6%)	31 (34.8%)		
Yes	123 (71.1%)	65 (77.4%)	58 (65.2%)		
**Psychiatric diagnoses** ^**1**^ **and assessment at FPI**					
Psychiatric diagnoses (DSM-IV Axel I^2^, II and/or III^3^), *n* (%)				0.000***	2.120 (1.798–2.499)^†^
No	16 (9.1%)	0 (0.0%)	16 (17.6%)		
Yes	159 (90.9%)	84 (100.0%)	75 (82.4%)		
Disorders usually first diagnosed in infancy, childhood, or adolescence, *n* (%)	37 (21.1%)	23 (27.4%)	14 (15.4%)	0.052	2.074 (0.985–4.366)^†^
Schizophrenia and other psychotic disorders, *n* (%)	41 (23.4%)	40 (47.6%)	1 (1.1%)	0.000***	81.818 (10.888–614.802)^†^
Bipolar disorders, *n* (%)	6 (3.4%)	4 (4.8%)	2 (2.2%)	0.429^╒^	2.225 (0.397–12.476)^†^
Depressive disorders, *n* (%)	37 (21.1%)	22 (26.2%)	15 (16.5%)	0.116	1.798 (0.860–3.757)^†^
Anxiety disorders, *n* (%)	36 (20.6%)	11 (13.1%)	25 (27.5%)	0.019*	0.398 (0.182–0.871)^†^
Somatoform disorders, *n* (%)	3 (1.7%)	1 (1.2%)	2 (2.2%)	1.000^╒^	0.536 (0.048–6.023)^†^
Sexual and gender identity disorders, *n* (%)	1 (0.6%)	1 (1.2%)	0 (0.0%)	0.480^╒^	0.477 (0.408–0.557)^†^
Impulse-control disorders not elsewhere classified, *n* (%)	1 (0.6%)	1 (1.2%)	0 (0.0%)	0.480^╒^	0.477 (0.408–0.557)^†^
Delirium, dementia, and amnestic and other cognitive disorders, *n* (%)	2 (1.1%)	2 (1.1%)	0 (0.0%)	0.229^╒^	0.474 (0.405–0.555)^†^
Personality disorders, *n* (%)	62 (35.4%)	21 (25.0%)	41 (45.1%)	0.006**	0.407 (0.214–0.774)^†^
Other relevant diagnoses, Axel III^2^, *n* (%)	8 (4.6%)	5 (6.0%)	3 (3.3%)	0.483^╒^	1.857 (0.430–8.020)^†^
Assessed intellectual level, *n* (%)	(*n* = 135)	(*n* = 59)	(*n* = 76)	0.701	0.102^††^
Clearly above average	13 (9.6%)	6 (10.2%)	7 (9.2%)		
Average	86 (63.7%)	37 (62.7%)	49 (64.5%)		
Clearly below average	26 (19.3%)	10 (16.9%)	16 (21.1%)		
Significantly below average	10 (7.4%)	6 (10.2%)	4 (5.3%)		

^#^The concept of a severe mental disorder is the Swedish legal term for when an offender according to a forensic psychiatric investigation is judged to fulfill criteria for compulsory forensic psychiatric care instead of a prison sanction. ^╒^Fischer’s exact test was the statistical test chosen to calculate the p value when the expected value in a fourfold table was less than five. The method chosen to calculate the effect size, indicated by a specific symbol as shown below, was depending on the statistical test we performed. ^†^Odds ratio, 95% confidence interval. ^††^Cramer’s V. *p < 0.05; **p < 0.01; ***p < 0.001. Bold figures in cells. *Indicate significant difference at α-level corrected by the Bonferroni method (i.e., rounded to 0.001). ^1^Individuals could have more than one diagnosis. ^2^Excl. individuals diagnosed with only substance use disorders. ^3^Diagnoses at Axel III, relevant for the psychological functioning (DSM-IV), e.g., Fragile X syndrome, Frontal lobe syndrome, Temporal lobe epilepsy.

### 3.3. Substance use/abuse characteristics

Overall, at the time of the FPI, it was established that a majority of all offenders had shown signs of prior (65.3%) and/or present (57.5%) substance use/abuse (Table 3). A smaller proportion of the whole sample had previously been diagnosed with substance use disorders (25.1%), and undergone substance use/abuse treatment services (31.6%), while as many as 40.6% were diagnosed with substance use disorders at the FPI. Further, the SMD group had, compared to the no-SMD group, distinctly less often (though not significant according to the Bonferroni-corrected significance level) present substance use/abuse (44.2% vs. 68.9%; Cramer’s V 0.254), previous substance use disorders/diagnoses (16.7% vs. 33.0%.; OR 0.407 [0.198–0.837]), and received care for substance use (23.2 vs. 39.3%; OR 0.465 [0.239–0.906]). Furthermore, the odds of having been diagnosed with a substance use disorders/diagnoses at the time of the FPI were also less likely among the SMD group compared to the no-SMD group (27.4% vs. 52.7%; OR 0.338 [0.180–0.635]). Concerning the offenders with present or previous substance use/abuse, no significant differences between the SMD versus no-SMD group were found neither regarding the age of onset nor severe negative consequences.

**Table 3:** Substance use characteristics regarding women, with and without a severe mental disorder^#^ (SMD), who were charged with lethal or attempted lethal violence and examined according to a court ordered forensic psychiatric investigation in Sweden, carried out between 2000 and 2014.

	All offenders (*n* = 175)	SMD group (*n* = 84)	No-SMD group (*n* = 91)	*p* value (*χ*^2^)	Effect size
Previous substance use/abuse^1^, *n* (%)				0.099	0.192^††^
No	59 (34.7%)	35 (43.8%)	24 (26.7%)		
Yes, alcohol	17 (10.0%)	7 (8.8%)	10 (11.1%)		
Yes, psychoactive substances	18 (10.6%)	9 (11.3%)	9 (10.0%)		
Yes, alcohol & psychoactive subs	76 (44.7%)	29 (36.3%)	47 (52.2%)		
Present substance use/abuse^1^, *n* (%)				0.013*	0.254^††^
No	71 (42.5%)	43 (55.8%)	28 (31.1%)		
Yes, alcohol	25 (15.0%)	9 (11.7%)	16 (17.8%)		
Yes, psychoactive substances	14 (8.4%)	6 (7.8%)	8 (8.9%)		
Yes, alcohol and psychoactive subs	57 (34.1%)	19 (24.7%)	38 (42.2%)		
Debut/onset of alcohol, problematic use/abuse - age range, *n* (%)	(*n* = 97)	(*n* = 39)	(*n* = 58)	0.766	0.074^††^
Child, <15 years	51 (52.6%)	19 (48.7%)	32 (55.2%)		
Youth, 15–18 years	29 (29.9%)	12 (30.8%)	17 (29.3%)		
Adult, >18 years	17 (17.5%)	8 (20.5%)	9 (15.5%)		
Debut/onset of psychoactive substance use/abuse - age range, *n* (%)	(*n* = 87)	(*n* = 35)	(*n* = 52)	0.778	0.076^††^
Child, <15 years	24 (27.6%)	10 (28.6%)	14 (26.9%)		
Youth, 15–18 years	37 (42.5%)	16 (45.7%)	21 (40.4%)		
Adult, >18 years	26 (29.9%)	9 (25.7%)	17 (32.7%)		
Severe negative consequences^2^ of substance use/abuse, *n* (%)	(*n* = 81)	(*n* = 32)	(*n* = 49)	0.541	0.163^††^
No	11 (13.6%)	6 (18.8%)	5 (10.2%)		
1 severe consequence	4 (4.9%)	1 (3.1%)	3 (6.1%)		
2–3 severe consequences	17 (21.0%)	5 (15.6%)	12 (24.5%)		
>3 severe negative consequences	49 (60.5%)	20 (62.5%)	29 (59.2%)		
Previous diagnoses—substance use disorders, *n* (%)				0.013*	0.407 (0.198–0.837)^†^
No	131 (74.9%)	70 (83.3%)	61 (67.0%)		
Yes	44 (25.1%)	14 (16.7%)	30 (33.0%)		
Previous substance use/abuse treatment services, *n* (%)				0.023*	0.465 (0.239–0.906)^†^
No	117 (68.4%)	63 (76.8%)	54 (60.7%)		
Yes	54 (31.6%)	19 (23.2%)	35 (39.3%)		
Diagnoses at FPI—substance use disorders, *n* (%)				0.001***	0.338 (0.180–0.635)^†^
No	104 (59.4%)	61 (72.6%)	43 (47.3%)		
Yes	71 (40.6%)	23 (27.4%)	48 (52.7%)		

^#^The concept of a severe mental disorder is the Swedish legal term for when an offender according to a forensic psychiatric investigation is judged to fulfill criteria for compulsory forensic psychiatric care instead of a prison sanction. The method chosen to calculate the effect size, indicated by a specific symbol as shown below, was depending on the statistical test we performed. ^†^Odds ratio, 95% confidence interval. ^††^Cramer’s V. *p < 0.05; **p < 0.01; ***p < 0.001. Bold figures in cells. *Indicate significant difference at α-level corrected by the Bonferroni method (i.e., rounded to 0.001). ^1^Substance use/abuse refers to extended use of alcohol and/or psychoactive substances, some of which have been diagnosed with substance use disorders. ^2^E.g., substance use/abuse caused severe negative consequences in regard to family life (e.g., conflicts, separation); professional and employment situation (e.g., loss of job, periods of unemployment); led to social marginalization, loss of housing, and other profound social problems; criminality.

### 3.4. Crime characteristics

A majority of the offender group did not have a previous criminal record (59.5%), and the actual charge for most offenders consisted of some type of attempted lethal violence (i.e., attempted murder, attempted voluntary manslaughter, and conspiracy/instigation/preparation to attempted murder; 61.7%). Most of them had committed the crime on their own (89.0%), in a home (80.3%), and used sharp violence (62.3%). About half of the sample was at the time of their crime under the influence of some kind of substance (49.0%), and about 40% had previously abused the victim (39.5%), while a minority had attempted homicide-suicide (10.6%; Table 4). Regarding the victims, they were commonly adults (80.7%), single victims (78.3%), males (71.3%), and intimate partners/ex-intimate partners (37.7%). Close to half of the victims were under the influence of substances at the time of the crime (44.3%), had previously abused the offender (44.3%), and about one-third of the victims had abused the offender at the time of the crime (32.6%). Even if there were no significant differences between the groups in several aspects, the odds of having a previous criminal record were less likely among the SMD group compared to the no-SMD group (26.8% vs. 52.7%; OR 0.328 [0.173–0.622]). Furthermore, the SMD group was distinctly less likely (though not significant according to the Bonferroni-corrected significance level) than the no-SMD group to have been charged with lethal violence (i.e., murder, voluntary manslaughter, involuntary manslaughter by assault, infanticide, and conspiracy/instigation/preparation to murder; 28.6% vs. 47.3%; OR 0.447 [0.238–0.836]), and also less likely to have acted under the influence of any substance (33.4% vs. 63.0%; Cramer’s V 0.307). When looking at victims the SMD group showed a more mixed pattern compared to the no-SMD group regarding victim-offender relationships (Cramer’s V 0.343; though not significant according to the Bonferroni-corrected significance level) with especially fewer intimate partners/ex-intimate partners (23.8% vs. 50.5%), but with a higher frequency of children (biological/adopted; 21.4% vs. 9.4%), friends/acquaintances (20.2% vs. 15.4%) or strangers (13.1% vs. 4.4%). Thereto, this mixed pattern was illustrated by differences regarding the victim’s gender with fewer males (59.5% vs. 82.8%; Cramer’s V 0.311) in the SMD group compared to the no-SMD group. There were also some differences concerning where the SMD group less often had victims that were under the influence of substance (23.6% vs. 63.5%; Cramer’s V 0.437), that less often had abused the offender. Finally, the SMD group was also more likely (though not significant according to the Bonferroni-corrected significance level) to have attempted homicide-suicide at the time of the crime (17.4% vs. 4.1%; OR 4.912 [1.322–18.253]).

**Table 4:** Crime characteristics regarding women, with and without a severe mental disorder^#^ (SMD), who were charged with lethal or attempted lethal violence and examined according to a court ordered forensic psychiatric investigation in Sweden, carried out between 2000 and 2014.

	All offenders (*n* = 175)	SMD group (*n* = 84)	No-SMD group (*n* = 91)	*p*-value (*χ*^2^)	Effect size
Offender, previously registered criminality, *n* (%)				0.001***	0.328 (0.173–0.622)^†^
No	103 (59.5%)	60 (73.2%)	43 (47.3%)		
Yes	70 (40.5%)	22 (26.8%)	48 (52.7%)		
Offender, charged with lethal versus attempt to lethal violence, *n* (%)				0.011*	0.447 (0.238–0.836)^†^
No	108 (61.7%)	60 (71.4%)	48 (52.7%)		
Yes	67 (38.3%)	24 (28.6%)	43 (47.3%)		
Multiple victims, *n* (%)				0.518	1.267 (0.617–2.603)^†^
No	137 (78.3%)	64 (76.2%)	73 (80.2%)		
Yes	38 (21.7%)	20 (23.8%)	18 (19.8%)		
Multiple offenders, *n* (%)				0.279	0.583 (0.218–1.561)^†^
No	154 (89.0%)	77 (91.7%)	77 (86.5%)		
Yes	19 (11.0%)	7 (8.3%)	12 (13.5%)		
Victim-offender relationship, *n* (%)				0.002**	0.343^††^
Intimate partner/ex. intimate partner	66 (37.7%)	20 (23.8%)	46 (50.5%)		
Child (biological/adopted)	27 (15.4%)	18 (21.4%)	9 (9.9%)		
Other family member/relative	24 (13.7%)	12 (14.3%)	12 (13.2%)		
Friend/acquainted	31 (17.7%)	17 (20.2%)	14 (15.4%)		
Professional	9 (5.1%)	6 (7.1%)	3 (3.3%)		
Stranger	15 (8.6%)	11 (13.1%)	4 (4.4%)		
Other	3 (1.7%)	0 (0.0%)	3 (3.3%)		
Victim gender, *n* (%)				0.000***	0.311^††^
Male	124 (71.3%)	50 (59.5%)	74 (82.2%)		
Female	47 (27.0%)	34 (40.5%)	13 (14.4%)		
Both male and female	3 (1.7%)	0 (0.0%)	3 (3.3%)		
Victim age range, *n* (%)				0.082	0.171^††^
Child, <15 years	29 (17.0%)	19 (23.8%)	10 (11.0%)		
Youth, 15–18 years	4 (2.3%)	2 (2.5%)	2 (2.2%)		
Adult, >18 years	138 (80.7%)	59 (73.8%)	79 (86.8%)		
Methods of violence, *n* (%)				0.298	0.167^††^
Blunt violence	14 (8.0%)	5 (6.0%)	9 (9.9%)		
Sharp violence	109 (62.3%)	48 (57.1%)	61 (67.0%)		
Asphyxia	15 (8.6%)	10 (11.9%)	5 (5.5%)		
Firearms	6 (3.4%)	3 (3.6%)	3 (3.3%)		
Other^1^	31 (17.7%)	18 (21.4%)	13 (14.3%)		
Crime scene, in a home, *n* (%)				0.518	0.781 (0.368–1.655)^†^
No^2^	34 (19.7%)	18 (21.7%)	16 (17.8%)		
Yes	139 (80.3%)	65 (78.3%)	74 (82.2%)		
Offender, attempted homicide-suicide, *n* (%)				0.010**	4.912 (1.322–18.253)^†^
No	127 (89.4%)	57 (82.6%)	70 (95.9%)		
Yes	15 (10.6%)	12 (17.4%)	3 (4.1%)		
Offender, under the influence of substance, *n* (%)				0.002**	0.307^††^
No	81 (50.9%)	50 (66.7%)	31 (36.9%)		
Yes, alcohol	39 (24.5%)	12 (16.0%)	27 (32.1%)		
Yes, psychoactive substances	11 (6.9%)	5 (6.7%)	6 (7.1%)		
Yes, alcohol and psychoactive subs	28 (17.6%)	8 (10.7%)	20 (23.8%)		
Victim, under the influence of substance, *n* (%)				0.000***	0.437^††^
No	79 (55.6%)	52 (76.5%)	27 (36.5%)		
Yes, alcohol	52 (36.6%)	11 (16.2%)	41 (55.4%)		
Yes, psychoactive substances	2 (1.4%)	0 (0.0%)	2 (2.7%)		
Yes, alcohol and psychoactive subs	9 (6.3%)	5 (7.4%)	4 (5.4%)		
Offender, previously abused^3^ victim, *n* (%)				0.400	0.734 (0.356–1.510)^†^
No	75 (60.5%)	41 (64.1%)	34 (56.7%)		
Yes	49 (39.5%)	23 (35.9%)	26 (43.3%)		
Victim, previously abused^3^ offender, *n* (%)				0.000***	0.212 (0.102–0.437)^†^
No	78 (55.7%)	50 (74.6%)	28 (38.4%)		
Yes	62 (44.3%)	17 (25.4%)	45 (61.6%)		
Victim, abused^3^ offender at the time of the crime, *n* (%)				0.000***	0.175 (0.077–0.394)^†^
No	89 (67.4%)	59 (84.3%)	30 (48.4%)		
Yes	43 (32.6%)	11 (15.7%)	32 (51.6%)		

^#^The concept of a severe mental disorder is the Swedish legal term for when an offender according to a forensic psychiatric investigation is judged to fulfil criteria for compulsory forensic psychiatric care instead of a prison sanction. The method chosen to calculate the effect size, indicated by a specific symbol as shown below, was depending on the statistical test we performed. ^†^Odds ratio, 95% confidence interval. ^††^Cramer’s V. *p < 0.05; **p < 0.01; ***p < 0.001. Bold figures in cells. *Indicate significant difference at α-level corrected by the Bonferroni method (i.e., rounded to 0.001). ^1^E.g., strangulation, fire, poison, extreme temperature, neglect, drowning, and hit by a car. ^2^E.g., public place (outdoors or indoors), solitary outdoors, unknown. ^3^Abused, defined as physical, sexual, and/or verbal violence/aggression.

## 4. Discussion

In this study characterizing female offenders of lethal and severe violence with and without an SMD in a Swedish 15-year sample, both common and specific group features were found. However, no significant differences were found between the SMD versus no-SMD group regarding background and demographic factors or previous mental health and care presenting female offenders as an overall burdened group, and in that sense with similar challenges. However, significant differences were found between the SMD versus no-SMD group regarding psychiatric diagnoses, substance use/abuse, and crime characteristics. These commonalities and differences, discussed further below, have implications for future research on female offenders of lethal and severe violence, particularly concerning FPI praxis as well as treatment within forensic psychiatry and correctional institutions.

### 4.1. Background and demographics

The present findings substantiate previous research of an overall pattern of social marginalization and adverse experiences both during upbringing and adulthood for female offenders. Since no background and demographic differences were found between the SMD/no-SMD groups, the discussion concerning background and demographics will focus on the obtained prevalences for the sample as a whole. Specifically, a relatively low educational level among female offenders was seen in line with prior research (15, 19, 23, 27, 28). Furthermore, the results indicate that despite prior work and education, few were employed or active students at the time of their crime, which is also consistent with previous studies (18, 27, 29). However, the civil status of these women was evenly distributed between offenders living within close relations/married versus those who were singles/unmarried (most of whom had previous experience of intimate partner relations). This diversity also mirrors prior research on civil status among female offenders of lethal and severe violence, where some studies have found a dominance of married/within intimate partner relations while other studies have found an unmarried/single pattern. The dominance of married/within intimate partner relations has especially been found among female homicide offenders in general (6, 27, 29), but also among specific subgroups such as maternal filicide offenders with mental illness (20), while single civil status has been shown in subgroups with mental illness in general (23), prison samples (31, 34), or infant homicide^4^ offenders (19). This diversity likely mirrors differences in study samples, some using a broader sample and other more specific samples. For example, for obvious reasons, studies on intimate partner homicide (15, 16) have a higher prevalence of offenders within intimate partner relations/marriage. Further, there was also a high prevalence of adverse childhood experience and lifetime victimization within the total sample of female offenders, which likewise is consistent with prior research (20, 26, 27, 29, 31). In conclusion, regarding background factors, the results in the present study were in line with previous studies, and also merge previous disparate results. Furthermore, the results particularly emphasize the importance of considering adverse experiences in both preventive work (e.g., within psychiatric, somatic, and maternal and child healthcare as well as the social services and police) and risk management when working with female offenders of lethal and severe violence.

### 4.2. Mental health characteristics

The present study showed that a majority of the female offenders had previous psychiatric diagnoses, and psychiatric care experiences with, in many cases, attempted suicide, which is consistent with prior studies (20, 23, 27, 31). These findings appeared to have considerable negative effects on their everyday life and are probably indications of social marginalization (e.g., ability to work, sickness benefit). Despite the observed generally low educational level, in this study, both female offenders with SMD versus no-SMD had an intellectual level within the normal range. This is in line with previous results showing that a prison sample (i.e., which shares most characteristics with the no-SMD group) in which female homicide offenders performed within normal limits but below average (34). Further, it should be noted that approximately 20% of the whole sample in the present study were not cognitively assessed and the prevalence of IQ testing was even lower for the SMD group.

Moreover, in line with previous studies (20, 23), the SMD group unsurprisingly showed a higher prevalence of psychiatric diagnoses, in particular, psychotic disorders compared to the no-SMD group. The no-SMD group instead had a higher prevalence of personality disorders, which is in line with previous research on female lethal and severe violent offenders within prison samples (31, 34).

### 4.3. Substance use/abuse characteristics

Among the female offenders in the present study with substance use/abuse, most showed an age of onset during adolescence. This highlights the importance to identify substance use/abuse at an early age, consistent with previous research emphasizing how early substance abuse seems to advance the debut as well as increase the incidence of criminal behavior and violence (21). Despite the present findings showing a relatively high prevalence of substance use disorders assessed at the FPI, as well as previous substance use/abuse treatment services, the amount of previously assessed substance use disorders was relatively low compared to the ones assessed at the time of the FPI. This indicates that substance use disorders might have been underdiagnosed for these female offenders, leading to possible treatment interventions not being offered to a sufficient extent.

Regarding comparisons between the two groups, the no-SMD group more frequently had substance use diagnoses compared to the SMD group. These findings are consistent with prior research showing that a majority of female offenders have had experiences of substance abuse (22, 27, 34). This is also illustrated in a study on female intimate partner homicide offenders who often had substance abuse (18). In the present study, substance use/abuse were more prevalent in the no-SMD group. Though, as prior studies show a dominance of substance abuse also among female offenders within forensic psychiatric care (21, 26), it seems to be contradictory to the present finding that the SMD group had a smaller proportion of substance use disorders. Therefore, it could be relevant to consider the impact of the legislation in different countries, and in future research studies the effect of different legal practices when sentencing offenders with substance abuse and intoxication during their crime.

### 4.4. Crime characteristics

Overall, most of the victims of the female offenders were in a close relationship with the offender (i.e., partners, relatives, and acquaintances) consistent with previous studies (23, 38, 39, 46–48), most often as current or previous intimate partners of the offenders (41). Furthermore, consistent with prior research, the victim predominantly was an adult (40), where the crime took place within a home (20, 39), and the most common method used was sharp violence (42).

However, differing patterns were seen for the SMD versus the no-SMD group. The SMD group was more often charged with attempt to lethal violence, as had they more frequently attempted homicide-suicide at the time of the crime. Furthermore, the SMD group more often had victimized children (biological/adopted), friends/acquaintances, or strangers. Interestingly, for the SMD group, female victims were also more common than in the no-SMD group. Partly, the gender aspect could be explained by the fact that the SMD group has more child victims, and that there has been an overrepresentation of female gender among child victims according to previous research (6). These differences in victim characteristics might also be understood as a consequence of whom the offender has a close relationship. That is, maybe individuals that have a more severe mental disorder are closer and get support from other relatives than intimate partners. This is seen in the case of strangers and acquaintances as victims, where the offender might live more isolated and the offense is directed within less close relationships and toward people who becomes part of the psychotic world of the perpetrator. An example is a victim that happens to meet a potential offender suffering from paranoid delusions, where the victim is experienced as a threat by the offender. All in all, the SMD group appears to be a group with a more heterogeneous pattern when it comes to victims. Hence, in the case of offenders with an SMD, the general picture of male intimate partners as victims of female offenders should be questioned. The no-SMD group, on the other hand, were more likely to have a previous criminal record, have been charged with lethal violence, and have been intoxicated during their crime. Their victims were more likely to be a male intimate partner/ex-intimate partner, being intoxicated at the time of the crime, and having previously (and/or at the time of the crime) abused the offender. Some of these results, such as being intoxicated at the offense and the offender being abused by the victim are in line with prior research (18, 36). However, the present result showing that a majority in the no-SMD group had a previous criminal record is somewhat disparate to prior studies displaying a more limited criminal record for female offenders (37).

To summarize, various factors seem to have preceded and driven the violence committed within the SMD versus the no-SMD group. To illustrate typical examples of an SMD versus a no-SMD offender, the SMD offender more often commits the crime influenced by mental illness and a psychotic inner state, toward a victim that is in a close relationship to her but not always, and with a relatively high risk of an attempted homicide-suicide at the time of the crime (compared to the no-SMD group). Contrariwise, illustrating a typical no-SMD female offender, committing the crime within a relationship where aggression and violence are common, where both she and her partner, who commonly is the victim, are using substances and are intoxicated at the time, and in a situation that is experienced as threatening. In some cases, the female no-SMD offender also has a history of a criminal past potentially influencing her violent behavior. Moreover, the obtained results regarding background, demographics, and previous mental health (e.g., previous unemployment, victimization, mental health problems, and suicidal attempts; i.e., common for the sample as a whole), indicate that the most common offender, both within the SMD and the no-SMD group, probably is affected by previous adverse experiences, mental health issues including suicide attempts, and unemployment. However, although these more prevalent examples illustrate the most common patterns of the female offenders in each group (SMD vs. no-SMD), the more atypical patterns are also important to bear in mind though they emphasize the importance of doing individual assessments of each case both regarding prevention and risk management. For example, in several cases, the offenders had no experience of previous adverse experiences, nor abuse by the victim or intoxication at the time of the crime. Further, even if more seldom, in about 10% the offender acted together with another offender, and in approximately 20% of all cases there was more than one victim, and the same percentage were seen for a crime scene that took place outside of a home-like environment. These are examples of atypical female offenders illustrating the complexity behind their criminal acts, which is why we in the striving to develop assessment and treatment of these offenders need to combine general patterns with individual trajectories.

## 5. Limitations and further directions

This study includes the majority of female offenders committing lethal and severe violence in Sweden during the observation period, indicating generalizability to this group as a whole in Sweden. Despite this, the study cohort is nevertheless relatively small, thereby potentially limiting the ability to statistically distinguish differences between the SMD versus the no-SMD group. Additionally, the fact that the sample inclusion criteria were based on a classification due to prosecution rather than the verdict, is also important to note regarding generalization, which includes a broader spectrum of violent offenses other than lethal and attempted lethal classifications within the final verdict. Further, being a cross-sectional study, based on FPIs and court verdicts which are not primarily intended for research purposes, may have restricted the possibility to detect all relevant information of the variables studied, thus hampering the possibility to identify differences between the SMD/no-SMD groups. However, the comprehensive material included in the FPI, together with court verdicts, comprises (in Sweden) a unique source of information due to its considerable focus on offender characteristics from three theoretical/clinical perspectives (medical/psychiatric, psychological, and social). Nevertheless, accomplished homicide-suicide cases were not included in the data material and since female offenders with child victims appear to commit homicide-suicide more often compared to female offenders with adult victims, this aspect is assumed to diminish generalizability to child homicide more than for those with adult victims (6). Hence, this should imply a knowledge gap and a margin of uncertainty regarding the possibility of identifying risk factors for the SMD group, as they show a larger proportion of child victims, as well as attempted homicide-suicide. Furthermore, since female homicide-suicide offenders could not be included, the likelihood of finding potential results/group differences pertaining to severe depression might be relatively low. Additionally, another knowledge gap could be assumed regarding a small portion of potential offenders who have not undergone an FPI due to no indications of a severe mental disorder at the time of the crime. These female offenders would not be included in the data material, rendering an uncertainty regarding the likelihood of identifying risk factors for offenders with no or minor indications of mental disorders, that is associated with the no-SMD group. Probably, these female offenders are characterized by more pronounced antisocial features, and their crimes are possibly of a more premeditated art and thus loading on traditional criminal risk factors. In future research, this potential knowledge gap could hopefully be managed by focusing on female offenders of lethal and severe violence within the prison and probation services for whom no FPI has been conducted. Further, regarding mental health, it might be of interest to mention that in previous studies it has been argued that female offenders to a larger extent are assessed and diagnosed from a perspective where they are viewed as victims and/or mentally disturbed, compared to male counterparts (52). Taken together though, our findings demonstrate that the total group of female offenders generally do have mental health difficulties, and have been exposed to trauma, as well as problems in many areas of life. Generalizing the results of the present study to other parts of the world must be done with certain caution due to differences in legislation. However, since the legal concept of SMD shares central traits with many other western countries to a considerable extent (e.g., Not Guilty by Reason of Insanity), these results can also be informative in an international context (44, 45).

## 6. Conclusion

Overall, female offenders charged with lethal and severe violence who had undergone an FPI were generally characterized by a high prevalence of mental health issues and victimization. However, the female offenders who suffered from an SMD differed in several respects from those who did not fulfill the criteria for an SMD. The SMD group more often had psychotic disorders and broader/more varied victim relationships. Further, the no-SMD group had more previous registered criminality, personality- and substance use disorders, as well as more often intimate partners as victims. Overall, these results mainly support previous research on female lethal and severe violence, but also merge previous disparate results as well as nuances them. Studying differences between the SMD versus no-SMD group regarding mental health, substance use, and crime characteristics in more detail would be an important topic to dwell upon more deeply. Also, further research focuses on personality aspects and risk factors based on various assessment instruments, and long-term follow-up studies would be desirable. Obtaining detailed knowledge regarding female offenders of lethal and severe violence could lead to better-developed strategies, and to the allocation of resources within general healthcare to identify increased risk for a woman to commit such serious violent crimes. This may also aid the criminal justice system and forensic psychiatric care to develop suitable re-offending programs for these offenders. Hopefully, the present results may stimulate further research focus on female lethal and severe violence, both using qualitative and quantitative methods. Such continued research would further aid in constructing and developing preventive strategies and reoffending management for the, in so many ways, costly consequences of the serious crimes that this group commits, as well as take preventive action within healthcare institutions (e.g., maternal and child healthcare and psychiatric care), social services, and police authority.

## Data availability statement

The raw data supporting the conclusions of this article will be made available by the authors, without undue reservation.

## Ethics statement

The studies involving human participants were reviewed and approved by The Regional Ethical Board in Gothenburg (registration number: 788-14). Written informed consent from the participants’ legal guardian/next of kin was not required to participate in this study in accordance with the national legislation and the institutional requirements.

## Author contributions

KT, TN, and MHK formulated and designed the study. KT assessed/rated a majority of the research protocols, with assistance from TN and MHK. KT did all statistical analyses, and wrote the first draft of the manuscript. All authors have continuously given feedback on the manuscript and approved the final version.

## Funding

The study was financed by grants to TN from the Swedish state under the agreement between the Swedish Government and the county councils, the ALF-agreement (ALFGBG-819681), and to KT from the Local Research and Development Council of Gothenburg and South Bohuslän, grant number VGFOUGSB-869731.

## Conflict of interest

The authors declare that the research was conducted in the absence of any commercial or financial relationships that could be construed as a potential conflict of interest.

## Publisher’s note

All claims expressed in this article are solely those of the authors and do not necessarily represent those of their affiliated organizations, or those of the publisher, the editors and the reviewers. Any product that may be evaluated in this article, or claim that may be made by its manufacturer, is not guaranteed or endorsed by the publisher.

## Footnotes

^1^The research protocol guide can be obtained (in Swedish) from the first author.

^2^The protocol consisted of 644 variables in total.

^3^Severe negative consequences, e.g., substance abuse caused severe negative consequences in regard to family life (e.g., conflicts, separation); profession and employment situation (e.g., loss of job, periods of unemployment); led to social marginalization, loss of housing and other profound social problems; criminality.

^4^This is an example of the difficulties getting an overview of female homicide offenders due to the use of narrow but related concepts, such as Maternal filicide defined as "the murder of a child by its mother" and Infant homicide, in the study by Wilson et al. (19), defined as" the death of a child before the first birthday."
