# Supplementary material for: Lethal and severe violence: Characterizing Swedish female offenders with and without a severe mental disorder
Source: Front Psychiatry. 2023 Apr 6;14:1143936. doi: 10.3389/fpsyt.2023.1143936 (PMC10117968; doi:10.3389/fpsyt.2023.1143936)
Supplement: Supplementary file 1 [file Table_1.docx]

**Supplementary Table 1.** Interrater Correlation Coefficient (ICC)^1^ regarding variables concerning women, with and without a severe mental disorder^#^ (SMD), who were charged with lethal or attempted lethal violence and examined according to a court ordered forensic psychiatric investigation in Sweden, carried out between 2000-2014.

| Variables^2^ | ICC value^1^ | 95% Confidence Interval |
| --- | --- | --- |
| **Excellent = 1.000** |  |  |
| SMD at the time of the crime | 1.000 | 1.000-1.000 |
| SMD at the time of the FPI | 1.000 | 1.000-1.000 |
| Previous child- and adolescent institutional care | 1.000 | 1.000-1.000 |
| Previous substance use/abuse treatment services | 1.000 | 1.000-1.000 |
| Firearms (methods of violence) | 1.000 | 1.000-1.000 |
| Blunt violence (methods of violence) | 1.000 | 1.000-1.000 |
| Poison (methods of violence) | 1.000 | 1.000-1.000 |
| Strangulation (methods of violence) | 1.000 | 1.000-1.000 |
| Strangulation (by means of aids; methods of violence) | 1.000 | 1.000-1.000 |
| **Excellent > 0.900** |  |  |
| Psychiatric diagnoses, at FPI | 0.998 | 0.997-0.999 |
| Offender, age in years | 0.994 | 0.998-0.997 |
| Sharp violence (methods of violence) | 0.970 | 0.941-0.985 |
| Age at debut/onset of substance use/abuse | 0.959 | 0.872-0.987 |
| Mentally abused (offender <18 y) | 0.955 | 0.903-0.979 |
| Sexually abused (offender <18 y) | 0.944 | 0.869-0.975 |
| Substance abuse by guardians (offender <18 y) | 0.936 | 0.871-0.968 |
| Previous substance use/abuse | 0.935 | 0.864-0.969 |
| Substance use/abuse among cohabitants (offender >18 y) | 0.928 | 0.850-0.966 |
| Previous suicide attempt | 0.918 | 0.819-0.963 |
| Use other psychoactive substances than alcohol (present) | 0.917 | 0.832-0.959 |
| Not passed elementary school | 0.916 | 0.819-0.961 |
| Previous psychiatric care | 0.914 | 0.828-0.957 |
| Methods of violence | 0.913 | 0.827-0.956 |
| Previous psychiatric diagnoses | 0.911 | 0.817-0.957 |
| Offender previously physically abused the victim | 0.911 | 0.790-0.963 |
| Severe negative consequences of substance, problematic use/abuse | 0.911 | 0.512-0.983 |
| Abused, other than intimate-partner violence (offender >18 y) | 0.909 | 0.803-0.968 |
| **Good = 0.750-0.900** |  |  |
| Fire (methods of violence) | 0.883 | 0.766-0.941 |
| Sexually abused (offender >18 y) | 0.880 | 0.719-0.949 |
| Offender previously mentally abused the victim | 0.874 | 0.648-0.956 |
| Use alcohol (present) | 0.872 | 0.737-0.938 |
| Abused, intimate-partner violence (offender >18 y) | 0.859 | 0.694-0.935 |
| Violence/aggression within family (offender <18 y) | 0.858 | 0.697-0.934 |
| Offense classification/charged with (lethal vs. attempt to lethal) | 0.841 | 0.680-0.920 |
| Used other psychoactive substances than alcohol (previous) | 0.817 | 0.637-0.908 |
| Substance abuse among relatives and cohabitants (offender >18 y) | 0.802 | 0.572-0.908 |
| Abused (offender <18 y) | 0.775 | 0.488-0.899 |
| Asphyxia (by means of aids; methods of violence) | 0.766 | 0.519-0.886 |
| Abused (Offender >18 y) | 0.764 | 0.497-0.890 |
| **Moderate = 0.500-0.750** |  |  |
| Offender previously abused victim | 0.715 | 0.252-0.895 |
| Abused, other than primary family (offender <18 y) | 0.708 | 0.383-0.863 |
| Other methods of violence (methods of violence) | 0.579 | 0.162-0.792 |

^#^ The concept of a severe mental disorder is the Swedish legal term for when an offender according to a forensic psychiatric investigation is judged to fulfill criteria for compulsory forensic psychiatric care instead of a prison sanction.

^1^ For interrater reliability analyses intraclass correlation coefficient (ICC value: average measures) was used, with two-way mixed effects model, single rater type, and absolute agreement as definition.

^2^ Eight analyzed variables were not included in the table since the obtained value was either “Zero variance” (due to only “No” and “Do not know” answers, which in a few cases differed between the two raters), or .000 (for variables with limited information for the raters, and therefore mostly answered “No” and “Do not know”, but in a few cases with “Yes” answers). None of these variables were in themselves used in any analysis concerning presented results, but some of them have been added with other variables into overarching categories for analytic use (e.g., “Offender previously threatened victim with physical violence” within the overarching category “Offender previously abused victim”).
